# Occurrence and Distribution of Synthetic Organic Substances in Boreal Coniferous Forest Soils Fertilized with Hygienized Municipal Sewage Sludge 

**DOI:** 10.3390/antibiotics2030352

**Published:** 2013-07-17

**Authors:** Richard Lindberg, Kenneth Sahlén, Mats Tysklind

**Affiliations:** 1 Department of Chemistry, Umeå University, Umeå 90187, Sweden; E-Mail: mats.tysklind@chem.umu.se; 2 Department of Forest Ecology and Management, Swedish University of Agricultural Sciences, Umeå 90183, Sweden; E-Mail: kenneth.sahlen@ssko.slu.se

**Keywords:** sludge, soil, antibiotics, hormones, POPs

## Abstract

The occurrence and distribution of synthetic organic substances following application of dried and granulated (hygienized) municipal sewage sludge in Swedish boreal coniferous forests were investigated. Elevated concentrations of triclosan (TCS), polybrominated diphenyl ethers (PBDEs), and polychlorinated biphenyls (PCBs) were detected in the humus layer. Concentrations of ethinyl estradiol (EE2), norfloxacin, ciprofloxacin, ofloxacin (FQs), and polyaromatic hydrocarbons (PAHs) were not significantly influenced. Maximum concentrations in humus were as follows (in ng/g dry matter): TCS; 778; PBDEs; 25; and PCB7; 16.7. Fertilization did not alter the levels of the substances in mineral soil, ground water, and various types of samples related to air. Further research within this area is needed, including ecotoxicological effects and fate, in order to improve the knowledge regarding the use of sludge as a fertilizing agent. Continuous annual monitoring, with respect to sampling and analysis, should be conducted on the already-fertilized fields.

## 1. Introduction

According to the Swedish government in 2006, the growth of the Swedish forest should increase 20% the following ten years, by means of fertilization, in order to replace the use of fossil fuels [1]. A similar conclusion was made during the evaluation of the Forest Bill 2007 [2]. In addition, the usage of such fertilizers in forest land should increase. The most essential nutrient for growth is nitrogen, and forest fertilization with nitrogen based fertilizers has been done over a long period [3]. An increase in growth, in the range of 15–20 m^3^·ha^−1^, is possible with a nitrogen dose of 150 kg·ha^−1^. Today, approximately 60,000 ha is fertilized in Sweden each year. On withdrawal of whole trees and rejected tops and branches, a larger nutrient loss is expected via needles, in comparison to the traditional collection of timber and pulpwood. In addition, the losses in growth due to nitrogen deficiency may also follow thinning [4]. Today, wood ash is recommended in order to compensate for the nutrient output following collection of tops and branches [5]. However, the ash lacks nitrogen and may cause growth reductions in less fertile soils [6].

In Sweden, about 240,000 tons of municipal sewage sludge (dry matter, dm) are produced and, according to one of the Swedish environmental goals, at least 60% of phosphorus should be reused as fertilizer [7]. Sewage sludge contains all of the essential nutrients to prevent growth loss following withdrawal of whole trees, and rejected tops and branches. The nitrogen and the organic matter content in sludge contribute to a higher production and will improve the soil’s ability to maintain the nutrients. The use of dried granular sludge as a fertilizing agent has shown that growth increases of at least 50% can be obtained [8]. A significant portion of the nitrogen in the sludge is organically bound allowing higher doses, in comparison to nitrogen fertilizers, without leakage of nitrate via ground water. In North America positive effects were noticed for more than 15 years following fertilizing forests with sewage sludge [9]. It has also been shown that if sewage sludge is used as a nitrogen source, carbon emissions caused by the manufacturers of fertilizers will decrease [10]. 

Municipal sewage sludge does not only consist of nutrients, on the contrary, heavy metals and organic substances that may cause adverse environmental effects are also present. The levels of heavy metal levels are significantly lower in sludge in comparison to wood ash (e.g., only 10% cadmium), and if the nutritional compensation is made with sludge, the total load of metals would be lower. Numerous studies of the effects of heavy metals in the forest ecosystem have been made, and they conclude that it is unlikely that adverse environmental effects may occur in a practical application of forest fertilizing [11]. However, in terms of the organic contaminants, the knowledge regarding their fate in boreal coniferous forests, following fertilization with sewage sludge, is poor. Contaminants associated to municipal sewage sludge include: flame retardants and plasticizers, e.g., polybrominated diphenyl ethers (PBDEs) and polychlorinated biphenyls (PCBs); byproducts, e.g., polyaromatic hydrocarbons (PAHs); antibacterial agents in hygiene products, e.g., triclosan (TCS); and pharmaceutical residues such as fluoroquinolone antibiotics (FQs) and ethinyl estradiol (EE2) [12,13,14,15]. In Sweden, the use of PCBs was banned in 1972, but during the gradual phasing out, PCB could be present in imported closed systems, e.g., capacitors and transformers, during the following 20 years. From 1995 and forward, PCBs should not exist in any products or systems in Sweden [16]. The regulation of PAHs (European Community) comprises a sales ban if the total concentration of the eight prioritized PAHS is greater than 10 mg·kg^−1^ (1 mg·kg^−1^ for Benzo[a]pyrene) in tires or the oil for the manufacturing of tires [17]. Other sources of PAHs include car exhaust, the wearing down of tire and road materials, and petrol stations. A total ban of PBDEs within the European Union has not yet been initiated, with the exception of penta- and octabrominated diphenyls in 2004, and PBDE #209 in 2006 (not be used in electronic products). The use of TCS has been questioned worldwide and the Swedish Society for Nature Conservation requires a total ban on this substance in any consumer products (e.g., in toothpastes and textiles) due to its environmental hazards [18]. The Swedish Pharmaceutical Industry Association has launched a simple environmental classification (based on information from the pharmaceutical manufacturers) with the goal that all active pharmaceutical ingredients will have environmentally relevant information within five years [19]. Negative environmental impact of EE2 (used in combination birth control pills) is not excluded since ecotoxicological information is missing. FQs (used in treatment of conditions such as urinary tract infection) have been classified by some of the manufacturers as (ciprofloxacin only): may cause a negative environmental impact, potentially persistent, and not readily bioaccumulative.

In a Swedish screening study of five municipal sewage water treatment plants (STPs), the detection frequency of three FQs in digested dewatered sludge was 100% [13]. These substances have also been detected in hygienized sludge, *i.e.*, dried digested sludge (above 90% dm), at Umeå STP [20]. In an extensive study by the U.S. Environmental Protection Agency, 84 sludge samples from 74 sewage treatment plants were investigated with the aim to determine four PAHs, 11 flame retardants, 72 pharmaceuticals, and 25 steroids and hormones [21]. Seen in relation to the total number of sludge samples that were analyzed, the detection frequency was as follows (in percentages): semi-volatile organic compounds and PAHs, over 50%; brominated flame retardants, 100%; 12 pharmaceutical compounds (including ciprofloxacin, diphenhydramine, and triclocarban), approximately 95%; and nine steroids, approximately 95%. In general, the levels of TCS and FQs in sludge are in the mg·kg^−1^ dm range. The corresponding levels of PBDEs, PAHs, PCBs, and EE2 are in the ng kg^−1^ dm range [12,13,14,15,21].

Information on the levels of synthetic organic substances in humus and mineral soil in sludge fertilized forest soils are scarce. In arable land, subjected to sludge fertilization, levels of PCBs and PBDEs were elevated, and bioaccumulation in earthworms was detected [22]. It has been shown that FQs may accumulate in soil if fertilization occurs regularly [12,23]. In general, several different biochemical and toxic effects, such as interference in reproduction, mutagenicity, and carcinogenicity, may occur in organisms exposed to TCS, PBDEs, PCBs, and PAHs. The main question regarding FQs and TCS is if their presence in the environment gives rise to resistant strains of bacteria, which in turn can produce changes in the ecosystem. Negative environmental effects of hormones have been reported, *i.e.*, androgynous fish downstream of English STPs [24].

The aim of this study is to: (1) evaluate occurrence and distribution of selected synthetic organic substances in relevant matrices following application of dried and granulated municipal sewage sludge to boreal coniferous forest soils; and (2) perform an initial environmental risk assessment.

## 2. Results and Discussion

### 2.1. Levels of Synthetic Organic Substances in Granulated Sludge

The concentrations of the synthetic organic substances (see Table 1) in granulated sludge (in µg·kg^−1^ dm) were as follows. FQ tot 6920; TCS 1170; EE2 1,4; PCB-7 41; PBDE tot 56; PAH-L 13; PAH-M 770; and PAH-H 750. FQs and TCS had the highest concentrations in the granulated sludge used for fertilizing L1. The levels of these four substances in the sludge are similar to those previously reported [12,13,15,20,21,25]. The EE2 concentration was approximately three orders of magnitude lower than those of FQs and TCS, but still in close correlation to previous findings in municipal sludge [26]. In addition, the levels of PBDEs (PBDE #209 not included in the PBDE tot of granulated sludge), PAHs, and PCBs were similar to those reported from the US Environmental Protection Agency [21]. For both PAHs and PCB-7, levels are below the recommended guide values, 3,000 and 400 µg·kg^−1^ dm, respectively, suggested being used within agriculture (according to a national agreement) [27]. 

**Table 1:** The synthetic organic substances included in this study.

Substance	CAS *^a^*	M*w ^a,b^* (g mol-1)	Log *P ^a,c^*	p*K*_a1_*^a,d^*	p*K_a2_^a,e^*
*Antibacterial*					
Triclosan (TCS)	3380-34-5	289.54	5.34	7,8	-
*Antibiotics ^f^*					
Norfloxacin	70458-96-7	319.33	1.74	0.16	8.68
Ofloxacin	82419-36-1	361.37	1.86	5.19	7.37
Ciprofloxacin	85721-33-1	331.35	1.63	6.43	8.68
*Hormone*					
Ethinyl estradiol (EE2)	57-63-6	296.40	4.11	10.24	-
*PBDEs ^g^*					
PBDE 47	5436-43-1	485.79	6.68	-	-
PBDE 99	60348-60-9	564,69	7.31	-	-
PBDE 183	207122-16-5	722.48	8.19	-	-
PBDE 209	1163-19-5	959. 17	9.45	-	-
*PAHs ^h^*					
Acenaphtylene *^i^*	209-96-8	152.19	3.27	-	-
Acenaphthene *^i^*	83-32-9	154.21	3.73	-	-
Fluorene *^ j^*	86-73-7	166.22	4.32	-	-
Phenanthrene *^j^*	1985-01-08	178.22	4.55	-	-
Anthracene *^j^*	120-12-7	178.22	4.55	-	-
Pyrene *^j^*	129-00-0	202.25	5.00	-	-
Fluoranthene *^j^*	206-44-0	202.25	5.00	-	-
Benz[a]anthracene *^k^*	56-55-3	228.29	5.73	-	-
Chrysene *^k^*	218-01-9	228.29	5.73	-	-
Benzo[b]fluoranthene *^k^*	205-99-2	252.31	6.19	-	-
Benzo[k]fluoranthene *^k^*	207-08-9	252.31	6.19	-	-
Benzo[*a*]pyrene *^k^*	50-32-8	252.31	6.19	-	-
Dibenzo[*a,h*]anthracene *^k^*	53-70-3	278.35	6.91	-	-
Indeno[1,2,3,*cd*]pyrene *^k^*	193-39-5	276.33	6.65	-	-
Benzo[*ghi*]perylene *^k^*	191-24-2	276.33	6.65	-	-
*PCB-7 ^L^*					
PCB 28	7012-37-5	257.54	5.72	-	-
PCB 52	35693-99-3	291.99	5.83	-	-
PCB 101	37680-73-2	326.43	6.44	-	-
PCB 118	31508-00-6	326.43	6.77	-	-
PCB 138	35065-28-2	360.88	6.98	-	-
PCB 153	35065-27-1	360.88	7.04	-	-
PCB 180	35065-29-3	395.32	7.51	-	-

^a^ Reference [28]; ^b^ Molecular weight; ^c^ Octanol/water partition coefficient; ^d^ Acid dissociation constant of first proton; ^e^ Acid dissociation constant of second proton; ^f^ Fluoroquinolones (FQs); ^g^ Polybrominated biphenyl ethers; ^h^ Polyaromatic hydrocarbons; ^i^ Low molecular weight; ^j^ medium molecular weight; ^k^ high molecular weight, carcinogenic; ^l^ Indicator PCBs. The seven congeners usually analyzed, constitute 10%−30% of total PCB-content (in total 209 congeners).

### 2.2. Levels of Synthetic Organic Substances in the Humus Layer and Mineral Soil in the Small-Scale Experiment (L1)

The extraction efficiency for the FQs from humus layers was inadequate, although this basic extraction method is suitable for sludge [13,20] and mineral soil. Although modifications of the presented method were made, including various strengths of EDTA and basic (or acidic) additives, added amounts of FQ substances were not recovered, at least not chemically intact, from the humus layer. Similar problems regarding extraction of antibiotics, including FQs, from solids and humic material have been addressed [29,30]. This is most likely due to a strong association of FQs to humus components. In house experiments, investigating the phase distribution of ciprofloxacin in water/humus matter systems, showed similar results. If this assumption is valid, the humus layer might act as a trap, in turn preventing the FQs from reaching the mineral soil and the soil water. The FQs were below LOQ in all of the mineral soil samples. The frequency of detection of EE2 was also low and it was only above LOQ in the dried granulated sludge.

**Figure 1:**
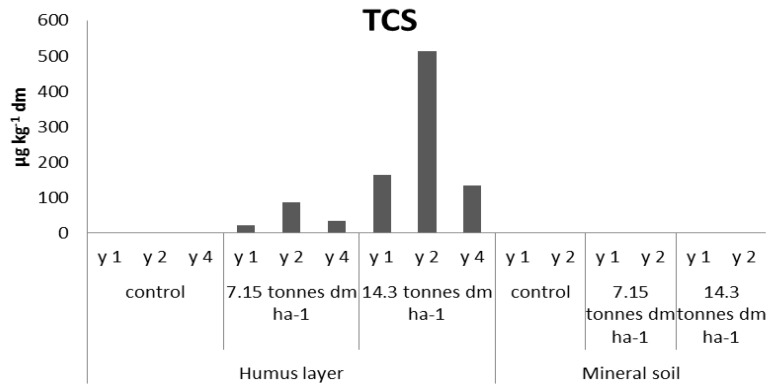
TCS levels in the small-scale experimental sites fertilized by hand (L1).

TCS was detected at both sites fertilized by hand with dried granulated sludge, and the highest levels (humus) were found on the site with the highest dose (164 and 513 µg·kg^−1^ dm), shown in Figure 1. In the following two years, the concentrations of TCS have increased approximately three times, most likely due to further degradation of the granule itself. Following a total of four years, the TCS concentration in the fertilized humus layers have lowered, indicating that degradation or removal processes have occurred. However, the levels are still in close correlation to the findings of the first year of sampling. TCS was below LOQ in the mineral soil, suggesting that this substance’s ability to reach underlying soil is limited, at least during the first two years following fertilizing.

**Figure 2:**
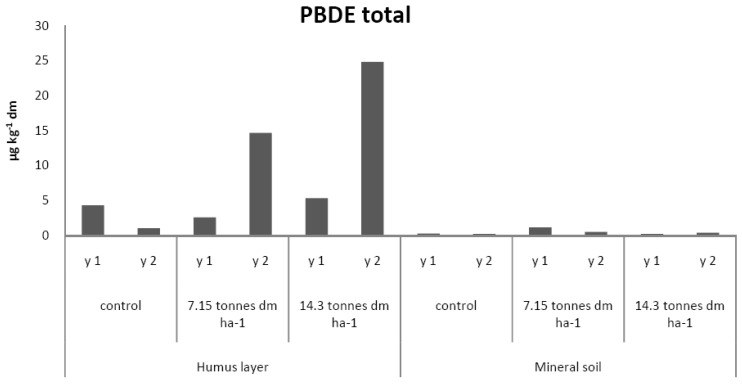
PBDE total levels in the small-scale experimental sites fertilized by hand (L1).

**Figure 3:**
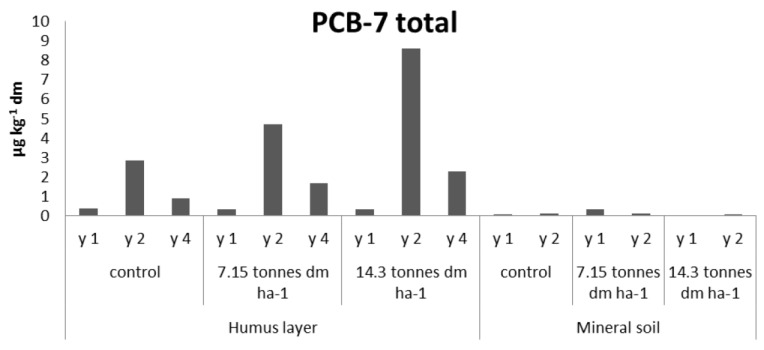
PCB-7 total levels in the small-scale experimental sites fertilized by hand (L1).

**Figure 4:**
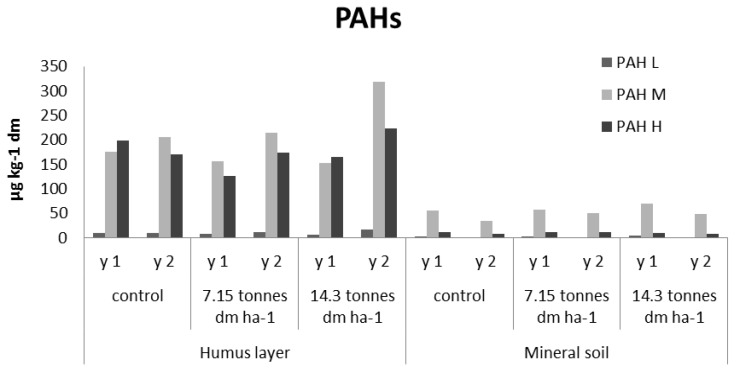
PAH levels in the small-scale experimental sites fertilized by hand (L1).

The presence of PBDEs, PCBs, and PAHs in forest soils, in comparison to FQs, TCS, and EE2, is different since atmospheric deposition may give rise to background levels. In order to assess an increase of the concentration of such substances in fertilized soils, their levels should exceed those found in the control sites. The PBDEs and PCB-7 have, in parity with TCS, elevated levels in humus of the fertilized sites and it is clearly demonstrated in year two (Figure 2, Figure 3, respectively). The concentrations of PBC-7 of the fourth year sampling of the humus layer are, as in the case of TCS, lower than the results of the second year of sampling. In the fertilized mineral soil samples, the levels of PBDEs are in close correlation to those found in the control sample. The corresponding levels of PCB-7 were below 0.4 µg·kg^−1^. Of the three groups of PAHs, none showed significant change following fertilizing, regardless of soil matrix, and instead, their levels were in close connection to those found in the controls, see Figure 4.

### 2.3. Levels of Synthetic Organic Substances in the Humus Layer and Mineral Soil in the Full-Scale Sites (L3-L4)

In Table 2, the results from the L3 and L4 sites are shown. FQs and EE2 were not found above their LOQ in any of the samples collected from full-scale sites (L3 and L4), including soil water (only FQs). TCS was present in all humus samples exposed to dried granulated sludge, and the highest concentration was seen in L4. Lower levels of TCS were seen in L3, but a positive result was obtained from a site not being fertilized. This could be explained by atmospheric deposition of TCS, however it is questionable if TCS is distributed in the environment via air. TCS was below LOQ in all but one of the mineral soil samples. PBDEs were present in all but one of the samples collected from the full-scale experimental locations (mineral soil sample from L4) and slightly elevated levels could be seen in the humus layer samples exposed to sludge. By comparison to the small-scale (L1) location, the levels of PBDEs in the full-scale (L3-L4) locations were slightly lower. In location L4 the concentrations of PCB-7, PAH-M, and PAH-H were approximately two to five times higher in the humus layer exposed to sludge compared to the control equivalents. Elevated levels of these three groups of synthetic organic substances could not be seen in the mineral soil. In addition, the results from the air sampling campaign (in L2) did not reveal any significant increases of the levels of the synthetic organic substances following full scale fertilizing by machine, regardless of the sample type (Table 3).

**Table 2:** Levels (µg kg^−1^ dm) of the synthetic organic substances in samples from the full-scale sites.

	L3 *^a^*								L4 *^b^*			
Substances	H *^c^*	H	H *^d^*	H *^d^*	M *^e^*	M	M *^d^*	M *^d^*	H	H *^d^*	M	M *^d^*
FQ tot	*^f^*	*^f^*	*^f^*	*^f^*	*^g^*	*^g^*	*^g^*	*^g^*	*^f^*	*^f^*	*^g^*	*^g^*
TCS	9	*^g^*	31	18	*^g^*	*^g^*	17	*^g^*	*^g^/^gh^*	778/69*^h^*	*^g^*	*^g^*
EE2	*^g^*	*^g^*	*^g^*	*^g^*	*^g^*	*^g^*	*^g^*	*^g^*	*^g^*	*^g^*	*^g^*	*^g^*
PBDE tot	0.16	2.1	0.67	3.3	0.03	0.20	0.35	*^g^*	0.001	0.07	0.08	0.06
PCB-7	2.77	3.36	2.69	4.53	0.09	0.10	0.69	0.08	5.79/2,9*^h^*	16.70/10,00*^h^*	0.10	0.14
PAH-L	11	8	1	*^g^*	*^g^*	*^g^*	*^g^*	*^g^*	12	21	2	1
PAH-M	215	205	4	289	2	4	9	1	196	525	16	15
PAH-H	212	216	2	293	2	1	5	1	234	1199	12	18

*^a^* Furuberget location, see Table 4; *^b^* Bäcksjön location, see Table 4; *^c^* Humic layer samples; *^d^* Fertilized site; *^e^* Mineral soil sample; *^f^* Not analyzed; *^g^* Below limits of quantification; *^h^* Results of the additional sampling conducted October 2011.

**Table 3:** The absolute amount (in ng) in the passive samplers (SPMD), levels in air (active air sampling) and in pine needles, in ng m^3 ^^−1^ and µg kg^−1^ wet weight, respectively.

Substances		SPMD *^a^*	SPMD *^b^*	Air *^a^*	Air *^b^*	Pine needles *^a^*	Pine needles *^b^*
FQ tot		*^c^*	*^c^*	*^c^*	*^c^*	*^d^*	*^d^*
TCS		*^c^*	*^c^*	*^c^*	*^c^*	*^d^*	*^d^*
EE2		*^c^*	*^c^*	*^c^*	*^c^*	*^d^*	*^d^*
PBDE tot		4.3	8.2	0.008	0.005	2.42	0.99
PCB-7		3.7	2.9	0.03	0.02	0.17	0.12
PAH-L		0	1	0.5	0.4	0.4	0.5
PAH-M		92	64	1.4	0.8	13	16
PAH-H		29	59	0.2	0.1	7	4

*^a^* Before fertilizing; *^b^* Following fertilizing; *^c^* Not analyzed; *^d^* Below limits of quantification.

### 2.4. Average Concentrations of the Synthetic Organic Substances in the Humus Layer

**Figure 5:**
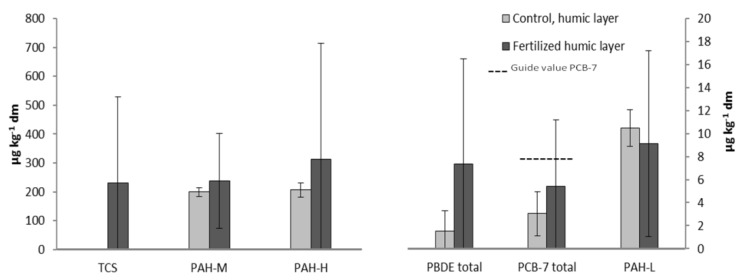
Average concentrations and standard deviations of the synthetic organic substances in control and fertilized humus samples.

The average concentrations and standard deviations of the synthetic organic substances were calculated, see Figure 5. The results were based on: humus samples, control and fertilized sites (n = 5 of each), and all of the locations (L1-L4) included in this study (the results of L1 were based on concentrations obtained from the year two sampling campaign). The average concentrations were, in all but one case, namely PAH-L, elevated in sites fertilized with dried and granulated sludge and the most significant increase were seen in TCS and PBDEs concentrations. The immense standard deviations are most likely attributed to the degradation of the sludge granule, and/or the synthetic substances, as the time between fertilizing and sampling range two to four years. In addition, the composition of the sludge collected from the Himmerfjärden STP during the different times (years) may vary. The Swedish guide value of PCB-7, concerning sensitive land use (8 µg·kg^−1^ dm), is also shown in Figure 5 and this value is equivalent to twice the background levels of PCB-7 obtained in this study [31]. In two of the fertilized locations, L1 year two and L4, obtained concentrations exceeding this value, suggesting that this soil is contaminated and restrictions, in terms of use and/or activity, should be practiced. Additional guide values, relevant to this study, are those of PAH-L (3 mg·kg^−1^ dm), PAH-M (3 mg·kg^−1^ dm), and PAH-H (1 mg·kg^−1^ dm), and in one of the sampling sites (location L4), the PAH-H concentration exceeded its guide value.

### 2.5. Environmental Risk Assessment

If humus, in fact, acts as a trap for the FQs, in combination with concentrations in mineral soil and ground water below the LOQ, the negative environmental impact of this group of substances may be negligible. However, it is unknown if the humus layer also disables the antibacterial properties of the FQs, in turn eliminating the risk of developing resistance strains of soil bacteria. Relevant studies investigating the effects of FQs on soil-living organisms are scarce, but several studies with concerns regarding the aquatic environment have been reported. The effects of ciprofloxacin on the bacterial community structure, with a focus on the mineralization of pyrene in marine sediment, yielded a calculated EC_50_ of 0.4 mg·kg^−1^ dm [32]. In other studies, plant uptake of FQs from the aquatic media were investigated and the conclusions were that this mode of action could not be excluded [33,34]. EE2 was only successfully quantified in the granulated sludge, and levels needed to develop an ecotoxicological response are close to the LOQs reported in this study [35]. Although many studies of the negative environmental impact of hormones exist, focus is attributed to organisms present in the aquatic media. Information regarding degradation of EE2 in soil matrices and its uptake in plants and bioaccumulation is missing. The risk associated with PAHs and fertilizing forest soils with granulated sludge, is most likely low since elevated levels of the PAHs, in contrast to the levels already present in the soil due to atmospheric deposition, could not be seen. 

The environmental risk, associated with fertilization of granulated sludge, was considered possible for TSC, PCB-7, and PBDEs, since the levels of these three groups of substances were significantly elevated in humus exposed to sludge (in comparison the equivalent controls). In addition, the concentrations of the toxic, bioaccumulative, and persistent PCB-7 exceeded the established guide value for contaminated soil in two cases (in humus of location L2 year two and L4). Although guide values of PBDEs in soil matrices is missing, and relevant ecotoxicological, bioaccumulation, and degradation studies of TCS in the soil environment are missing, the potential risk of environmental effects should be considered. In this study the fertilized sites have been exposed to granulated sludge for a maximum of five years. The long-term effect is approximately 15 years when using granulated sludge and, seen in that perspective, it is not unlikely that the levels of TCS, PCB-7, and PBDEs (seen in the L1 location) will persist due to further degradation of the granule itself. 

In an EU report from 2001, regarding organic contaminants in sludge used in agriculture, the available information at that time was compiled [36]. In the report, it is considered that the sludge is an important resource in order to return nutrients to agriculture. However, since sludge is the alternative holding the highest amounts of organic pollutants, the use of sludge cannot lead to adverse effects. The uptake of synthetic organic substances (e.g., PAHs, PCBs, PBDEs) in plants from soil is considered to be low but the uncertainty about other substances is high. The input of organic substances must be lower than their output (degradation), thus, it is possible to regulate the dose of various types of sludge. PCBs were identified as one of the candidates that should be monitored closely due to their vast degradation times. The level of knowledge regarding pharmaceuticals (including hormones) was assessed as very low and a proposal was that a permanent (at least 30-year-old) study about the persistence of organic compounds should be implemented prior to final recommendations. Not mentioned in the report are that synergistic effects between various organic substances (including metals) may occur and this phenomenon is not identified in toxicity tests targeting individual substances.

## 3. Experimental

### 3.1. Substances of Interest

The substances prioritized in this study can be seen in Table 1. The substances represent traditional persistent organic pollutants (PCBs, PAHs), as well as emerging environmental hazards (PBDEs and pharmaceuticals). The following substances are grouped and their individual concentrations are summed: FQs, PAH-L (low molecular weight), PAH-M (medium molecular weight), PAH-H (high molecular weight), PCB-7 (indicator PCBs), and PBDEs, see Table 1. 

### 3.2. Sludge Fertilized Forest Soils and Sampling

Samples were collected at four different sites in northern Sweden, see Table 4 for WGS84 coordinates and location characteristics. One of the sites (L1) was a small-scale experiment specifically prepared for this study. L1 was divided into three small test sites (10 × 10 m) and each site was fertilized by hand to ensure maximum control of the given dose. The dose of the dried granulated sludge (Himmerfjärden STP, Stockholm, Sweden) added to the three sites corresponded to (in tons dm·ha^−1^): 0 (control); 7.15; and 14.3. The three test sites were fertilized in September 2007 and the sampling was conducted in November of 2008 and 2009. Additional sampling of the humus layers, focusing on TCS and PCB-7, was performed during October 2011. From the top humus layers, five samples were collected and pooled during 2008, 2009, and 2011. From the mineral soil, five samples were collected and pooled during 2008 and 2009, at a depth of approximately 30 cm. This strategy was performed at all of the three sampling sites at L1. In addition, the granulated sludge used to fertilize L1 was collected 2008. 

**Table 4:** Information regarding the sampling locations.

Location	Lat. *^a^*	Long. *^a^*	Altitude (m)	Vegetation	Trees	Age *^b^*(years)
(L1) Snårberget	66,269039°	23,099030°	146	Bilberry	Pines	48
(L2) Räktjärvberget	66,153964°	22,898668°	130	Bilb./Ling.	Pines	46
(L3) Furuberget	66,545295°	22,571772°	95	Lingonberry	Pines	60
(L4) Bäcksjön	63,944599°	20,410820°	70	Bilberry	Pines	70

*^a^* According to WGS84; *^b^* Age is determined by counting the annual rings on an increment core, taken at 1,3 m height, with an addition of 10 years for the trees in order to reach 1,3 m height.

Active air sampling was conducted in L2 24 h before and 24 h during/following fertilizing by machine (in August 2008). Pine needles were collected two weeks before and two weeks following fertilizing. Passive air sampling, by means of semi-permeable membrane devices (SPMDs), was conducted three weeks before and three weeks following fertilizing. 

Humus and mineral soil (down to 30 cm depth) were gathered by pooling five subsamples (5 × 100 g) located in the middle, and in the four corners of a 2,000 m^2^ square, within L3. The sampling was made two years following fertilizing by machine (August 2006). The doses of the granulated sludge were 0 (control) and 19.8 tons dm·ha^−1^. 

Humus and mineral soil (at 30 cm depth) were gathered by pooling five subsamples (5 × 100 g) located in the middle and in the four corners of a 2,000 m^2^ square, within L4. The sampling was made five years following fertilizing by hand (Spring 2003). Five humus layer samples were also collected and pooled in October 2011. In addition, a soil water sample was also collected by the use of lysimeters from the control site and from the fertilized site. The doses of granulated sludge were 0 (control) and 13.6 tons dm·ha^−1^. 

### 3.3. Chemical Analysis

Prior to extraction of humus (cut to a maximum of 2 cm) and mineral soil samples, stones, and sludge granules were removed if needed. 

Fluoroquinolones (FQs): Isotopic labeled ciprofloxacin (^13^C_3_, ^15^N) was added to samples (2 g portions) of whole pine needles, mineral soil, and granulated sludge, to an amount of 1,000 ng per sample. The samples underwent liquid/solid extraction during 1 h with the use of 50% 0.1 M EDTA and 50% triethylamine (5%) in methanol/water (1:1). The sample extracts were spun at 5,000 rpm during 20 min and 1 mL of the supernatant was subjected to instrumental analysis. To the filtrated (0.45 µm) and acidified (pH 3) soil water samples (200 mL), 500 ng of the isotopically labeled ciprofloxacin was added and the samples were subjected to solid phase extraction (SPE, Isolute ENV+). The eluent was 5% triethylamine in methanol and the sample extracts were evaporated and re-dissolved in acidified (pH 3) acetonitrile/water (5/95). The sample extracts of the solid and aqueous phases were injected and analyzed by liquid chromatography electrospray tandem mass spectrometry (LC-ESI-MS/MS, LCQ DUO, Thermo Finnigan) [13]. The limits of quantification (LOQ) of solid and aqueous samples were 10 µg·kg^−1^ dm and 5 ng·L^−1^, respectively. 

Triclosan (TCS): Isotopic labeled triclosan (^13^C) were added (100 ng) to 2 g portions of of pine needles, humus layer, mineral soil, and granulated sludge samples. The samples were extracted 30 min in 15 mL methanol assisted by ultrasonication. The sample extracts were evaporated to approximately 1 mL and spun at 10,000 rpm during 20 min. The sample extracts were injected and analyzed by LC-atmospheric pressure photo ionization-MS/MS (TSQ Ultra, Thermo Electron) and the LOQ was 2.5 µg·kg^−1^ dm.

Ethinyl estradiol (EE2): Isotopic labeled EE2 (^13^C_2_) were added (500 ng) to 2 g portions of of pine needles, humus layer, mineral soil, and granulated sludge samples. The samples were extracted 30 min in 50 mL hexane/acetone (1:1) assisted by ultrasonication. The sample extracts were evaporated to dryness following extraction and re-dissolved in 10 mL acidified. The extracts were passed through SPE (Oasis HLB) before derivatization (silylation). The final sample extracts were injected and analyzed by gas chromatography high-resolution mass spectrometry (GC-HRMS, Micromass Ultima Autospec Ultra, (Waters Corp.). The LOQ was 1.2 µg·kg^−1^ dm.

Polyaromatic hydrocarbons (PAHs), polychlorinated biphenyls (PCBs), and polybrominated biphenyl ethers (PBDEs): Isotopic labeled analog representatives within each group were added to filter and polyurethane foam (from the active air sampling device), SPMD extracts and to 10 g portions of pine needles, humus layer, mineral soil, and granulated sludge. The samples were soxhlet extracted during 24 h in toluene, with the exception of pine needles that were extracted with dichloromethane and assisted by ultrasonication. The extracts were passed through multisilica (sulphuric acid, neutral, sodium hydroxide) and fluorisil, and the samples were injected and analyzed by GC-HRMS (Micromass Ultima Autospec Ultra, Waters Corporation). The LOQs (in ng kg^−1^) were: PCB 0.1; PBDE 1; and PAH-L 10; PAH-M 20; and PAH-H 40.

## 4. Conclusions

This study has shown that following fertilizing with dried granulated sludge, elevated levels of synthetic organic substances (mainly TCS, PBDEs, and PCB-7) occur in the humus layer. The correlation between the dose of the sludge used and the obtained concentrations were stronger in the small-scale experimental sites (100 m^2^), fertilized by hand, in comparison to the full-scale sites (several hectares), fertilized by machine. In some locations, the levels of PCB-7 slightly exceeded the guide value set by the Swedish Environmental Protection Agency (8 µg·kg^−1^). The guide value was only exceeded for sludge application rates above the proposed application rate (10 tons·ha^−1^) during forest fertilization in practical scale. Elevated levels of the synthetic organic substances were not found in the mineral soil, soil water, various types of samples related to air, or pine needles. Continuous monitoring of the synthetic organic substances should be conducted on the already fertilized locations. Even earlier experiments (if possible, more than 20 years ago) of forest soils, in which fertilization occurred with municipal sewage sludge, should be identified in order to improve the information regarding the levels, time trends, half-lives, and distribution of these substances in humus and mineral soils.
